# Prostate Abscess Associated With Severe Constipation: A Rare Case

**DOI:** 10.7759/cureus.80395

**Published:** 2025-03-11

**Authors:** Keisuke Suzuki, Shigetoshi Nishihara, Mako Sakakibara, Hitoshi Yoshida, Kenji Dohi

**Affiliations:** 1 Department of Emergency, Critical Care and Disaster Medicine, Showa Medical University School of Medicine, Tokyo, JPN; 2 Department of Medicine, Division of Gastroenterology, Showa Medical University School of Medicine, Tokyo, JPN

**Keywords:** constipation, infectious complications, ischemic reperfusion, older patient, prostate abscess

## Abstract

Severe constipation can cause ischemic bowel disease and other complications in older adults. However, its association with the formation of prostate abscesses has rarely been reported. An 80-year-old male recently presented to our center with syncope, dehydration, and constipation. Contrast-enhanced computed tomography (CT) revealed fecal impaction and a hypodense lesion in the prostate. Despite no initial elevation in inflammatory markers, the patient later developed recurrent hypotension. Follow-up CT confirmed a prostate abscess and rectal reperfusion. The absence of urinary white blood cells suggested a non-ascending urinary tract infection. Antibiotic therapy with ceftriaxone resulted in recovery. Severe constipation may contribute to prostate abscess formation via rectal ischemia and bacterial translocation. Early recognition and management of constipation are crucial to prevent severe complications in older patients. Further studies are warranted to clarify the specific pathway of this atypical infection.

## Introduction

Constipation is a common condition that may affect >10% of the adult population [1]. Chronic constipation is a prevalent condition that severely affects quality of life, with mild digestive difficulties being the most common symptom [2]. However, caution is advised because fecal impaction can cause ulcers, bleeding, perforation, and other serious complications in older adults. Severe constipation can exert systemic effects and has been linked to conditions such as ischemic bowel disease [3]. However, our search on PubMed revealed no previous reports in the literature regarding the associations between severe constipation and the formation of prostate abscesses.

Prostate abscesses are typically associated with ascending urinary tract infections or their hematogenous spreading, particularly in immunocompromised patients or those with diabetes [4]. We recently encountered a case that highlights a unique clinical scenario wherein severe constipation appeared to precipitate an ischemic reperfusion injury in the rectum, ultimately leading to the development of a prostate abscess. Understanding this atypical pathway may provide further insights regarding the potential complications associated with untreated severe constipation and the importance of timely interventions.

## Case presentation

**Figure 1:**
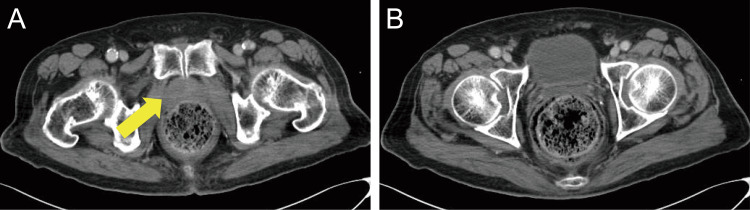
Contrast-enhanced CT performed on day 1 showing (A) a slightly lower-density area visible on the right side of the prostate (arrow) with no contrast effects seen in the surrounding region and (B) a significant quantity of fecal matter noted in the patient’s rectum CT: computed tomography

**Figure 2:**
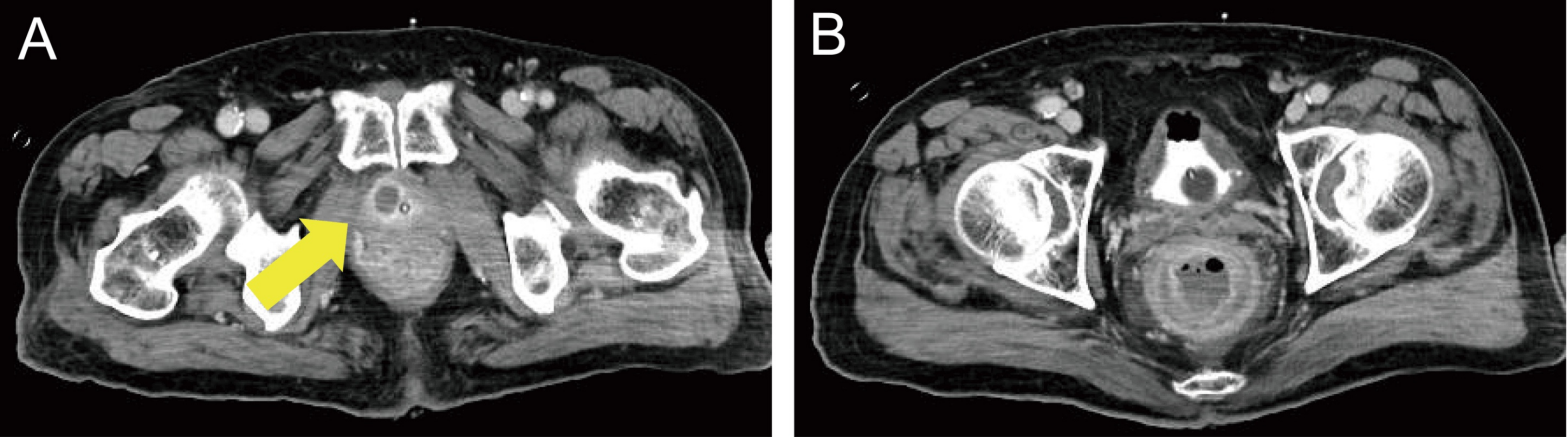
Contrast-enhanced CT performed on day 2 showing (A) a low-density area with a contrast effect seen surrounding the 15 × 12 mm region (arrow) and (B) the fecal mass expelled after which edema and a visible contrast effect were noted in the rectal wall CT: computed tomography

An 80-year-old male presented to the emergency department after experiencing an episode of syncope, and he reported having had no bowel movement for the past three days. He had no urological symptoms, such as dysuria or frequency. On arrival, his blood pressure was 76/47 mmHg, indicating hypotension. Other vital signs included a heart rate of 62 bpm, a respiratory rate of 29/min, a body temperature of 35.6°C, SpO₂ of 91% (r/a), and a Glasgow Coma Scale of E4V4M6. Following the administration of 1500 mL of intravenous fluids at the emergency department, his blood pressure rose to 110/65 mmHg, leading to the conclusion that the syncope was caused by hypovolemia. Contrast-enhanced computed tomography (CT) revealed fecal impaction within the rectum and a fuzzy low-density area in the prostate (Figure 1); however, blood tests showed no elevation in inflammatory markers (Table 1). The patient was diagnosed with dehydration and constipation and was admitted to the hospital following manual disimpaction of the fecal mass. During hospitalization, the patient experienced recurring episodes of hypotension. On day 2, blood tests revealed an elevated D-dimer level of 68.88 µg/mL and a C-reactive protein level of 4.91 mg/dL (Table 2). The marked elevation of D-dimer raised concerns about the possibility of thrombosis, while the elevated CRP suggested the presence of an infectious focus. Therefore, contrast-enhanced CT was performed, which revealed significant edematous wall thickening and misty mesentery around the rectum, suggesting ischemic-reperfusion injury (Figure 2). However, no evidence of thrombosis was observed. A well-defined abscess with ring enhancement had also developed in the prostate (Figure 2). Notably, no white blood cells were detected in the patient’s urine (Table 3), inconsistent with the typical findings of an ascending urinary tract infection. Combined with the patient’s history of several days without a bowel movement and imaging findings indicating severe fecal impaction leading to ischemic-reperfusion injury, we strongly suspected a connection between the prostate abscess and constipation. The patient was treated with ceftriaxone as an antibiotic for one week, which led to the normalization of both his laboratory and imaging findings.

**Table 1:** Laboratory data on day 1 WBC: white blood cell, Hb: hemoglobin, Plt; platelet, BUN: blood urea nitrogen, Cre: creatinine, AST: aspartate aminotransferase, ALT: alanine aminotransferase, CRP; C-reactive protein; PT-INR, prothrombin time-international normalized ratio; APTT, activated partial thromboplastin time

Parameter	Test result	Reference range
WBC (/μL)	5,800	3,900-9,700
Hb (g/dL)	13.7	13.4-17.1
Plt (×10^4^/μL)	21.7	15.3-34.6
BUN (mg/dL)	20.5	9.0-21.0
Cre (mg/dL)	1.11	0.60-1.00
AST (U/L)	16	5-37
ALT (U/L)	11	6-44
CRP (mg/dL)	<0.04	<0.30
PT-INR	1.02	0.90-1.10
APTT	22.2	24.0-34.0
D-dimer (μg/dL)	4.75	<1.00
Lactate (mmol/L)	2.02	0.6-1.4

**Table 2:** Laboratory data on day 2 WBC: white blood cell, Hb: hemoglobin, Plt: platelet, BUN: blood urea nitrogen, Cre: creatinine, AST: aspartate aminotransferase, ALT: alanine aminotransferase, CRP: C-reactive protein, PT-INR: prothrombin time-international normalized ratio, APTT: activated partial thromboplastin time

Parameter	Test result	Reference range
WBC (/μL)	10,000	3,900-9,700
Hb (g/dL)	13.7	13.4-17.1
Plt (×10^4^/μL)	18.3	15.3-34.6
BUN (mg/dL)	27.8	9.0-21.0
Cre (mg/dL)	1.15	0.60-1.00
AST (U/L)	20	5-37
ALT (U/L)	11	6-44
CRP (mg/dL)	4.91	<0.30
PT-INR	1.18	0.90-1.10
APTT	34.5	24.0-34.0
D-dimer (μg/dL)	68.86	<1.00
Lactate (mmol/L)	2.52	0.6-1.4

**Table 3:** Urinalysis data on day 2 No white blood cells were detected in the patient’s urine. HPF: high power field

Parameter	Test result	Reference range
Appearance	Clear	Clear
Protein	+/-	-
Ketones	-	-
Bilirubin	-	-
Urobilinogen	1+	-
Blood	2+	-
Red blood cells	5-9/HPF	<5/HPF
White blood cells	1-4/HPF	<5/HPF
Leukocyte esterase	-	-
Bacteria	-	-

## Discussion

This case demonstrates an atypical sequence of events wherein severe constipation contributed to ischemic-reperfusion injury in the rectum and the subsequent formation of a prostate abscess.

Prostate abscess is a rare condition thought to occur mainly due to ascending urinary tract infection. It is significantly more common in patients with diabetes or those who are immunocompromised [4]. Treatment may require antimicrobial therapy and drainage; however, the condition can lead to sepsis and even death if it worsens [4]. It is often difficult to distinguish prostatitis from other conditions, so CT is useful for diagnosis and follow-up [5]. In this case, urine leukocytes, which are positive in almost all patients with prostate abscesses, were not detected. Therefore, we suspected the abscess was caused by infection via a route other than the urethra, such as constipation.

The following three mechanisms are thought to be involved in the pathophysiology of this sequence of events. Severe fecal obstruction can cause blood flow disorders in the rectal mucosa. Fecal stones and hard stools in the colon may lead to pressure necrosis of the intestinal wall, resulting in fecal peritonitis. If the condition progresses, it has been reported to cause rectal perforation [6]. Ischemia-reperfusion injury may also play a role, as inflammation and bacterial migration can occur following the manual induction of defecation. This type of injury is strongly associated with inflammation [7]. The effect on the prostate should also be considered, as bacteria may migrate from the rectum to the prostate, leading to abscess formation. The rectum and prostate are particularly susceptible to such bacterial migration due to their close anatomical proximity.

This case study is limited in that there was no way to prove whether the patient’s constipation or prostate abscess formation occurred first. The extent to which a causal relationship exists also remains unclear. However, if the prostate abscess preceded the constipation, likely, urological symptoms such as increased inflammation, fever, perineal discomfort, and dysuria would have also been noted. Therefore, we concluded that the constipation likely occurred first, followed by the formation of the prostate abscess.

This case suggests the importance of managing chronic constipation, particularly in older adults. Primary measures that can be taken to address constipation include lifestyle improvements such as adopting healthier dietary and exercise-related practices [2]. If such approaches fail, drug-based and even surgical therapies may be considered in some cases. However, it has been shown that surgical treatment for chronic constipation does not always lead to high patient satisfaction [8]. It is possible to significantly reduce the risk of complications associated with constipation by appropriately controlling defecation. It is particularly appropriate to consider all available options and proceed optimally.

## Conclusions

This case underscores the importance of considering constipation as a potential risk factor for severe complications, such as the formation of prostate abscesses. Early identification and proper management of constipation are crucial to preventing serious complications, especially in older adults. Further research is warranted to explore the relationship between bowel dysfunction and pelvic infections.
